# ROS-Induced Endothelial Dysfunction in the Pathogenesis of Atherosclerosis

**DOI:** 10.14336/AD.2024.0309

**Published:** 2024-03-08

**Authors:** Ruiyi Yan, Xiao Zhang, Wenlong Xu, Jiayao Li, Yixin Sun, Shengyan Cui, Ran Xu, Wenjing Li, Liqun Jiao, Tao Wang

**Affiliations:** ^1^Eight-year Medical Doctor Program, Peking Union Medical College Hospital, Chinese Academy of Medical Sciences and Peking Union Medical College, Beijing, China.; ^2^Department of Neurosurgery, Xuanwu Hospital, Capital Medical University, Beijing, China.; ^3^Sir William Dunn School of Pathology, University of Oxford, Oxford, United Kingdom.; ^4^China International Neuroscience Institute (China-INI), Beijing, China.; ^5^First Hospital, Peking University, Beijing, China.; ^6^Laboratory of Computational Biology and Machine Intelligence, National Laboratory of Pattern Recognition, Institute of Automation, Chinese Academy of Sciences, Beijing, China.; ^7^School of Artificial Intelligence, University of Chinese Academy of Sciences, Beijing, China.; ^8^Department of Interventional Neuroradiology, Xuanwu Hospital, Capital Medical University, Beijing, China.

**Keywords:** oxidative stress, atherosclerosis, cardiovascular diseases, biomarkers, antioxidants

## Abstract

Various signaling pathways are regulated by reactive oxygen species (ROS), which are radical oxygen intermediates under normal physiological conditions. However, when the buffering capacity of antioxidant enzymes is exceeded by the accumulation of ROS, oxidative stress, and endothelial cell dysfunction occur, which have been recognized as key contributors to the development of atherosclerosis. In this review, an overview is provided on mechanisms underlying ROS generation in endothelial cells and the involved regulatory pathways. Further, we discuss the ROS induced endothelial cell dysfunction and its relationship with atherosclerosis. Current knowledge on ROS-induced endothelial impairment is presented, characterized by decreased NO bioavailability, intracellular dysfunction and ox-LDL accumulation. Furthermore, biomarkers such as oxidative products of lipid, protein, and nucleotide are discussed as measurements for ROS levels. Novel interventions targeting oxidative stress are listed as potential pharmacotherapies in clinical practice. In conclusion, this review presents a systematic analysis of the mechanisms underlying ROS generation and elucidates how manipulation of these mechanisms can safeguard endothelial cell function.

## 1. Introduction

Cardiovascular diseases (CVDs), such as angina, myocardial infarction, and ischemic stroke, are the leading causes of morbidity and mortality worldwide [1, 2]. Atherosclerosis is a chronic inflammatory disease that plays a pivotal role in the development of these conditions [3]. It is characterized by the formation of intimal plaques and accumulation of cholesterol in arterial walls [4]. Atherosclerosis involves fat deposition accompanied by macrophages and a fibrotic layer comprising smooth muscle cells, connective tissue, and leukocytes [5].

The vascular endothelium, consisting of a monolayer of flattened endothelial cells arranged on the inner surface of blood vessels [6], contributes extensively to maintain vascular homeostasis by regulating vasoactive mediators, cell adhesion, enzymatic buffering against reactive oxygen species (ROS), lipoprotein metabolism, and the migration and proliferation of smooth muscle cells. Following exposure to the risk factors of atherosclerosis, such as low-density lipoprotein (ox-LDL), blood pressure, and smoking, dysfunction of the vascular endothelium serves as the initial stage in the development of vascular diseases and leads to the earliest detectable changes in the life cycle of an atherosclerotic lesion [7, 8].

Imbalance between radical production and antioxidant defense system is the primary cause of oxidative stress, which is characterized by excessive generation of ROS and oxidized ox-LDL, is considered instrumental in the progression of atherosclerosis-related cardiovascular disease [9]. ROS induces endothelial dysfunction by impairing intracellular milieu, compromising NO production in endothelium, and increasing intravascular permeability, ultimately contributing to the development of atherosclerosis.

Therefore, the utilization of novel ROS-based therapies may provide a rational therapeutic strategy for improving endothelial dysfunction and preventing the development of atherosclerosis. Despite the extensive literature available on antioxidants and atherosclerosis, less attention has been given to an in-depth comprehension of the mechanisms generating ROS. This review aims to integrate fundamental molecular concepts with potential clinical applications. By comprehensively summarizing the pivotal role of oxidative stress in vascular endothelial dysfunction during the progression of atherosclerosis, and correspondingly evaluating therapeutic approaches targeting endothelial ROS, this article offers novel solutions specifically tailored for ROS-induced atherosclerosis.

## 1. Definition of ROS and the mechanism of ROS production

### 1.1. Definition of ROS

**Figure 1.**
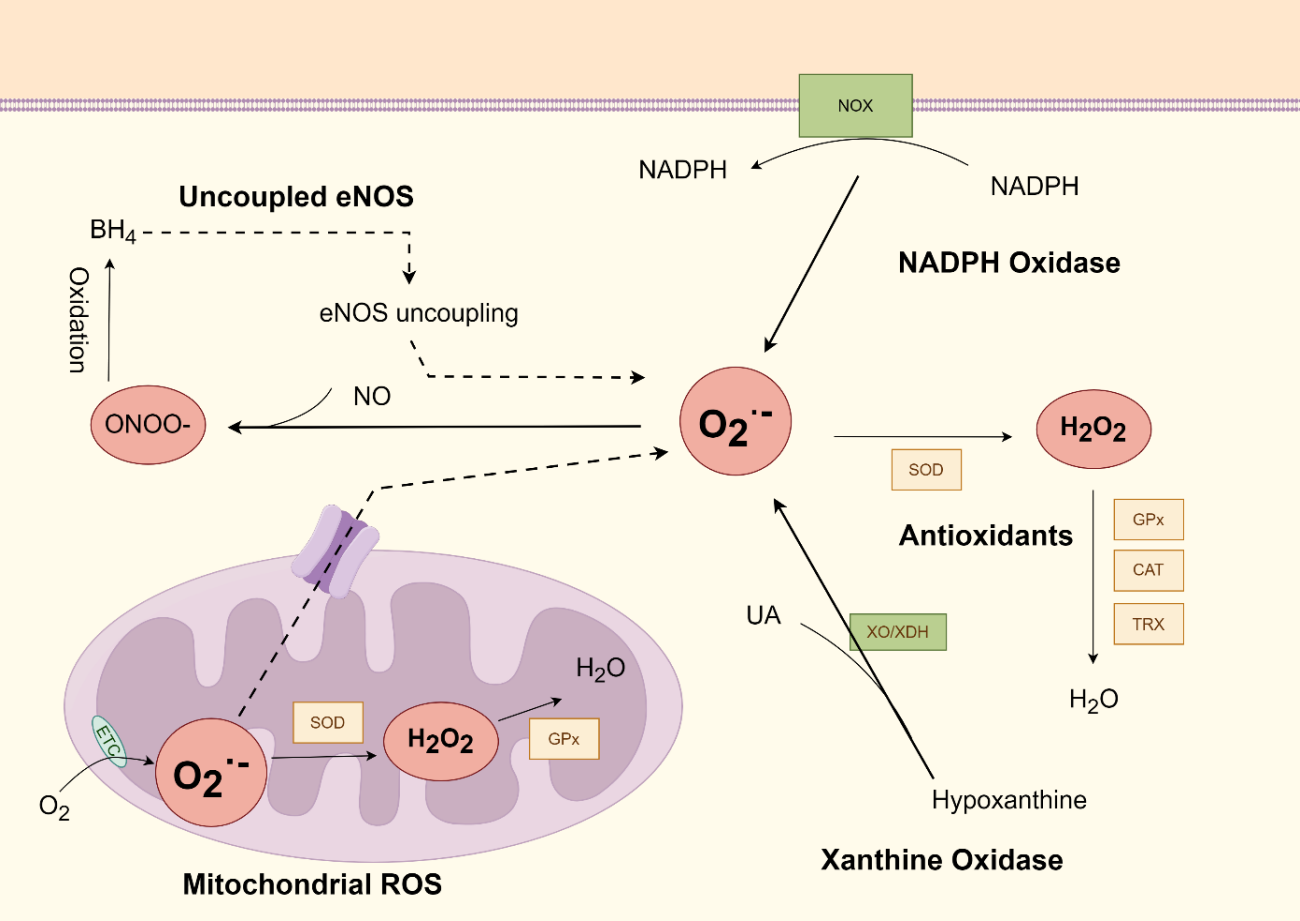
**ROS generation and removal in endothelial cells (By Figdraw)**. The main sources of ROS include mitochondrial electron transport chain, NADPH oxidase, Xanthine Oxidase, and uncoupled endothelial nitric oxide synthase (eNOS), which lead to increased generation of superoxide (O2^•-^). Antioxidant enzymatic systems including Superoxide dismutase (SOD), catalase (CAT), and glutathione peroxidase (GPx), involve the reduction process of reactive oxygen species. Abbreviations: ROS, Reactive oxygen species; H_2_O_2_, Hydrogen peroxide; ONOO^-^, Peroxynitrite; NO•, Nitric oxide; XO, Xanthine oxidase; XDH, Xanthine dehydrogenase; ETC, Electron transport chain.

According to the current understanding, oxidative stress can be defined as: “The excessive production of reactive oxygen species/nitrogen species (ROS/RNS) that cannot be counteracted by the action of antioxidants” [10].ROS/RNS species are represented by superoxide (O_2_·), hydroxyl (·OH), hydrogen peroxide (H_2_O_2_), singlet oxygen (1/2 O_2_), lipid peroxyl radicals, nitric oxide (NO), and peroxynitrite (OONO^-^) [11](Fig. 1).

### 1.2. Double-edged sword of ROS

Under physical conditions, the equilibrium between oxidation and reduction reactions maintains the stability of the internal environment. Physiological concentrations of ROS can regulate a variety of signaling pathways, regulating processes like inflammation, proliferation, and apoptosis. Elevated ROS can be beneficial in some situations, for example, notably in the macrophage when killing pathogens [12].

However, the imbalance between the ROS/RNS and antioxidant may trigger a cascade of responses in the cell, causing damage to macromolecules such as lipids, proteins, and DNA. In addition, high level of ROS production could lead to further ROS generation, causing ROS-induced ROS release, which promotes numerous chronic diseases [13]. Specifically, for the progression of atherosclerosis, oxidative stress is believed to be the key risk factor [14].

### 1.3. Production and regulation of ROS

Essentially, the generation of ROS occurs through several enzyme systems present in the vascular system. Endothelial cells and smooth muscle cells within the vascular wall possess the ability to produce a substantial amount of ROS via groups of enzymes, including nicotinamide adenine dinucleotide phosphate (NADPH oxidases), xanthine oxidase, the mitochondrial electron transport chain, and uncoupled endothelial nitric oxide synthase (eNOS) [12, 15]. In general, these enzymes facilitate electron transfer from their respective substrates to oxygen molecules, thereby regulating ROS level.

Importantly, there are cross-regulatory mechanisms exist between these pro-oxidant systems [16]. For instance, NADPH oxidase can produce initial ROS, which then trigger the activation of secondary sources of ROS [17]. Therefore, the primary, NADPH-derived ROS is postulated to be the “kindle” that activates the production of downstream source of ROS.

### 1.4. Antioxidant enzymatic systems

Endogenous antioxidants function as checkpoints to avoid the untoward consequences of ROS, which is accomplished by eliminating oxidants or decreasing the production of ROS. Superoxide dismutase (SOD), catalase (CAT), and glutathione peroxidase (GPx) are the main enzymes involved in ROS metabolism [18].

SOD catalyzes the dismutation of O_2_-radical into oxygen and H_2_O_2_, which is subsequently degraded by other antioxidant enzymes [19]. In humans, there are three isoforms of SOD based on their cellular localization. SOD1 is a soluble enzyme present in the cytoplasm and mitochondrial intermembrane space, while SOD2 is located in the mitochondrial matrix and SOD3 exists in the extracellular space [20]. The reductive function of SOD relies on sufficient downstream enzymes; otherwise, it can exacerbate oxidative stress due to H_2_O_2_ generation [21].

Catalase is a tetramer of four polypeptides, converting H_2_O_2_ to oxygen and water by four porphyrin heme iron groups. The inhibited expression and activity of catalase at the transcriptional level was revealed in the pathological environment of atherosclerosis [22]. From another point of view, catalases can ameliorate the state of peroxide accumulation and alleviate the atherosclerosis in high-fat diet mice models [21]. A recent experiment utilized hypercholesteremic mice with matched genotypes either harboring or lacking a single copy of the human catalase gene (CAT^Tg/0^ or CAT^0/0^). The findings revealed a notable reduction in oxidative stress, collagen deposition, and fiber thickness specifically observed in CAT^Tg/0^ mice [23]. Therefore, based on the findings from in vitro and animal model experiments, the overexpression of catalase may confer protective effects within the cardiovascular system.

Similar to catalase, GPx is an antioxidant enzyme that can reduce H_2_O_2_ to water and numerous other peroxides to their corresponding products. Similarly, Trx system can also reduce H_2_O_2_ and other target proteins. Studies in mice model show that the downregulation of Trx and GPx causes endothelial dysfunction and increases pro-atherogenic events [24, 25].

## 2. Vascular endothelial dysfunction

### 2.1. Definitions of endothelial dysfunction

The vascular endothelium consists of a monolayer of endothelial cells arranged on the inner surface of blood vessels that serve as a selectively permeable interface regulating fluid and macromolecule transport between circulation and tissues [26]. As our understanding of the endothelium has evolved over time, its metabolic functions have been increasingly recognized including regulation of vasoactive mediators, enzymatic buffering against ROS, metabolism of lipoproteins, and remodeling extracellular matrix components [27]. Therefore, when stimulated by certain inflammatory cytokines or bacterial products, endothelial cells undergo coordinated gene activation resulting in changes across many important functional domains.

Vascular endothelial dysfunction is characterized by an imbalance in the production of vasodilator and vasoconstrictor factors, resulting in a pro-thrombotic and pro-atherosclerotic phenotype. Thus, in the broad sense, endothelial dysfunction encompass various nonadaptive alterations, manifests as leakage from blood vessels, elevated ROS production, secretion of proinflammatory cytokines, increased expression levels for surface adhesion markers, and decreased NO production [8].

### 2.2. Association between endothelial dysfunction and atherosclerosis

**Figure 2.**
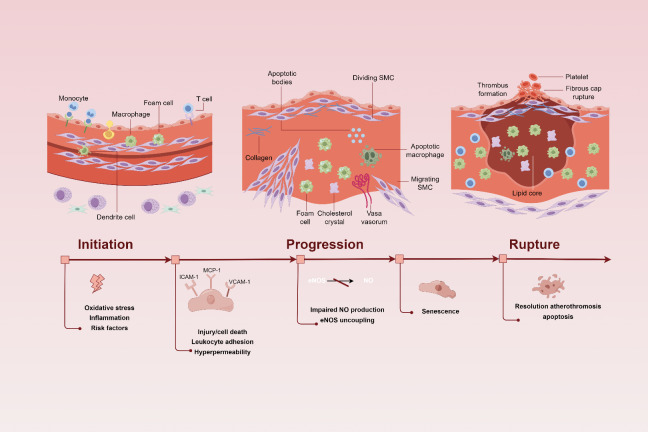
**Association between endothelial dysfunction and atherosclerosis (By Figdraw)**. In lesion-prone regions of the arterial vasculature, risk factors like proinflammatory agonists, oxidized lipoproteins (ox-LDL), as well as biomechanical stimulation leads to endothelial activation. These stimuli result in a coordinated program of genetic regulation within the endothelial cell, increasing the cell surface expression of adhesion molecules and secretion of membrane-associated chemokines. Together, these events foster the selective recruitment of leukocytes, which become resident in the subendothelial space, causing the progression of atherosclerosis. In advanced stages of atherosclerosis, plaque rupture seems to be a consequence of endothelial cell apoptosis, with localized endothelial denudation and the triggering of thrombus formation.

After exposure to the drivers of atherosclerosis, endothelial cells become activated, leading to the earliest detectable changes in the life cycle of an atherosclerotic lesion [7, 8] (Fig. 2). Once activated, endothelial cells express various adhesion molecules including ICAM-1, MCP-1, VCAM-1, P-selectin, and E-selectin, which selectively recruit circulating monocytes to the intima where they differentiate into macrophages and internalize modified lipoproteins to form foam cells [7, 28]. Activated endothelial cells and macrophages synthesize multiple chemokines and growth factors that subsequently act on adjacent smooth muscle cells inducing their proliferation and extracellular matrix synthesis, ultimately resulting in fibromuscular plaque formation [24, 28]. Additionally, developing lesions contain abundant inflammatory cells that further promote endothelial cell dysfunction progression. In advanced stages of atherosclerosis, increased interactions between platelet and endothelium was observed, indicating heightened endothelial dysfunction as a consequence of endothelial cell apoptosis [29]. These findings suggest that endothelial cell dysfunction is an important factor contributing to the development of atherosclerosis.

On the other hand, the environment of atherosclerosis also promotes the progress of endothelial cell dysfunction. This has been confirmed by in vitro experiments [30] where exposure to disturbed flow induces turnover and aging of ECs along with increased oxidative stress, altered cell shape as well as tissue remodeling of cytoskeletons and intercellular junction proteins similar to what is observed in vulnerable areas of arterial vasculature in vivo [31]. These observations suggest that different hemodynamic forces may constitute local risk factors for the occurrence of endothelial cell dysfunction within atherosclerosis thus promoting each other's phenotypic progression forming a vicious cycle [32].

## 3. Mechanism of oxidative stress induced endothelial dysfunction in AS

## 3.1 Sources of ROS in vascular endothelium

### 3.1.1. NADPH oxidase

NADPH dependent oxidoreductases (Nox enzymes) are expressed both in infiltrating monocytes and in resident cells in the vascular wall, whose primary function is to catalyze the production of superoxide from oxygen and NADPH. Nox enzymes participate in numerous crucial physiological processes, including cell growth [33], host defense and cellular signaling [34-36]. ROS produced by NOX participate in the proliferation, migration, and differentiation of EC, specifically including the xidative inactivation of tetrahydrobiopterin (H4B), the conversion of xanthine dehydrogenase (XDH) to XO by oxidation of the sulfhydryl residue and mitochondrial DNA damage [37, 38]. Thus, when Nox enzymes are under misadjustment, they can contribute to a wide range of severe pathologies, including atherosclerosis, hypertension, and cancer.

Nox enzymes are multi-subunits complexes, which contain two membrane-bound subunits and several cytosolic regulatory subunits, producing superoxide form molecular oxygen using NADPH as the electron donor [39]. NADPH oxidases family has seven isoforms, identified to include Nox1-5, Duox1, and Duox2 in humans. Among these isoforms, three of them are expressed in the vascular wall of mice: Nox1 and Nox4 in smooth muscle cells (VSMC) [40, 41], while Nox2 and Nox4 predominantly in endothelial cells [42, 43]. In addition, all of the Nox enzymes synthesize O_2_^-^ initially, but Nox4 is able to rapidly convert O_2_^-^ to H_2_O_2_ and emit it in terms of outcome [44]. Thus, while other Nox enzymes has been shown to serve as primary sources of ROS in ECs, Nox4 might have a protective role in the vasculature [45]. Besides, NOX3 is mainly expressed in the inner ear and has not been considered as key factor in vascular damage [46]. DUOX1 and DUOX2 are enrolled in immune response process and cell differentiation, both being expressed in the stomach, lungs, and thyroid, though their functions in the vascular system are not well understood [47].

### 3.1.2. Uncoupled eNOS

In endothelial cells, the eNOS enzyme serves as the primary source of NO, acting as powerful vasodilator by inducing relaxation of VSMCs. Also, NO function as an unconventional and highly reactive signaling molecule that regulates gene transcription, VSMC proliferation and attachment of leukocyte to endothelial cells [20]. The generation of NO by eNOS requires l-arginine and molecular oxygen (O_2_), along with the cofactors like tetrahydrobiopterin (BH4), NADPH, and flavin derivates [48]. Depletion of these essential molecules in conditions of ROS leads to uncoupling of eNOS, resulting in excessive production of O_2_^-^ instead of NO [49]. Then, superoxide reacts with NO to form OONO^-^, an unstable molecule that rapidly oxidizes BH4 while reducing the bioavailability of NO [48]. Consequently, eNOS becomes uncoupled and generates superoxide, further exacerbating the uncoupling process.

### 3.1.3. Xanthine Oxidases

Xanthine oxidase (XO), a form of xanthine oxidoreductase, is responsible for catalyzing the oxidation process of xanthine to uric acid, resulting in the generation of superoxide and hydrogen peroxide [50]. Another constitutively expressed xanthine dehydrogenase (XDH) generate superoxide under hypoxic conditions using NAD^+^ as an electron acceptor [51]. Uric acid is generated during XO reactions and plays a role in oxidative processes. Under normal physiological conditions, uric acid exhibits antioxidant properties by scavenging peroxynitrite, generating nitric oxide derivatives, or modulating ROS production through the Nrf2 pathway [52, 53]. However, an imbalance between synthesis and excretion of uric acid may result in hyperuricemia leading to inflammation and dysfunction within endothelial cells [52].

### 3.1.4. Mitochondrial Electron Transport Chain

Mitochondria is essential in cellular ATP generation, while also serving as a source of mitochondrial ROS (mtROS) that function as crucial signaling molecules in endothelial cells. MtROS is indispensable for the regulation of various cellular responses, encompassing stress response mechanisms [54-56]. The formation of mtROS occurs when electrons ‘spill’ onto oxygen from mitochondrial proteins located earlier on in the electron transport chain, predominantly manifesting as O_2_^-^ and H_2_O_2_. Stringent control over mtROS production by the electron transport chain is imperative to prevent oxidative damage to essential cellular processes [57]. Elevated levels of superoxide precipitate mitochondrial swelling and subsequent membrane rupture, leading to cytochrome c release, which triggers caspase cascades and ultimately instigates cell apoptosis [58]. Mitochondrial dysfunction represents a primary cause underlying excessive ROS generation and impaired EC functions.

## 3.2. Pathways Regulated by ROS

### 3.2.1. NF-κB

Initially identified as a DNA-binding protein in activated B cells, the transcription factor nuclear factor-κB (NF-κB) regulates gene expression in various cellular processes [59]. There has been growing evidence suggesting that the excessive generation of ROS, particularly H_2_O_2_, can directly or indirectly activate the NF-κB pathway. Firstly, H_2_O_2_ modifies NF-κB heterodimers and inhibits its upstream kinases such as IKK, leading to NF-κB activation and prevention of I-κB degradation [59]. Additionally, ROS promotes cytokine expression like TNF-α in endothelial cells as prototypical activators of the NF-κB pathway [60].

Under conditions of oxidative stress, NF-κB may have both anti-and prooxidative roles. It induces the expression of several antioxidant molecules that counteract ROS effects such as MnSOD, Ferritin Heavy Chain, Glutathione S-transferase pi (GSTP1), Metallothionein-3, and GPx-1 [59]. Alternatively, the NF-κB pathway can also have a prooxidant role by inducing genes like NADPH oxidase NOX2 subunit gp91phox, which switches endothelial cells to proinflammatory and pro-coagulatory phenotypes with increased thrombotic potential [61].

### 3.2.2. Nrf2

The nuclear factor erythroid 2-related factor 2 (Nrf2) is a transcription factor that forms inactive complexes with Kelch-like epichlorohydrin-related proteins (Keap1) under normal physiological conditions. Upon exposure to oxidative stress or other pathological stimuli, the cysteine residue of Keap1 undergoes modification, leading to Nrf2 phosphorylation and subsequent release from the complex. Subsequently, Nrf2 translocates to the nucleus where it interacts with antioxidant response elements (AREs), thereby promoting the transcription of a series of antioxidant enzyme, such as heme oxygenase (HO-1), SOD, and GSH-Px [62]. In addition, Nrf2 activation is associated with the induction of mitochondrial antioxidant enzymes such as Trx reductase-2 and GSH peroxidase. The Nrf2-Keap1-ARE signaling pathway is considered the most prominent pathway involved in maintaining cellular redox homeostasis [63]. In addition to Keap1, various protein kinases within the PI3K-Akt signaling pathway can phosphorylate Nrf2 and participate in its transcriptional regulation [64, 65].

HO-1, which serves as the rate-limiting enzyme in both the transformation and degradation of hemoglobin, is one of the main antioxidant enzymes activated by Nrf2 [66, 67]. Studies have shown that the Nrf2/HO-1 signaling can be activated by a variety of antioxidant factors including Glutaredoxin 2, abrogated oxidative stress in endothelial cells cultured *in vitr*o [68]. Thus, Nrf2/HO-1 pathway might exhibit antioxidative potential in protecting vascular endothelial cells from oxidative stress-induced injury [67, 69].

### 3.2.3. Sirtuins

Sirtuins are NAD^+^-dependent deacetylases that regulate critical cellular processes, and their increased expression and activity have been associated with enhanced cardiovascular function [70]. Among the seven sirtuin isoforms (SIRT1-7), SIRT1 exhibits particularly high expression in endothelial cells of arteries, veins, and capillaries [71]. It has been demonstrated that SIRT1 makes tremendous contributions in regulating endothelial function and mitigating premature aging induced by ROS, which is attributed to eNOS deacetylation [71, 72]. On the other hand, SIRT6 is predominantly localized within the nucleus and exerts its regulatory influence through chromatin remodeling [73], thereby playing a crucial role in maintaining telomere integrity via histone H3 lysine 9 (H3K9) deacetylation [74]. Notably, during H_2_O_2_-induced senescence, there is a decline in SIRT6 levels, and the overexpression of SIRT6 partially counteracts this process [75].

### 3.3. ROS induces vascular endothelial dysfunction through disturbing intracellular environment

**Figure 3.**
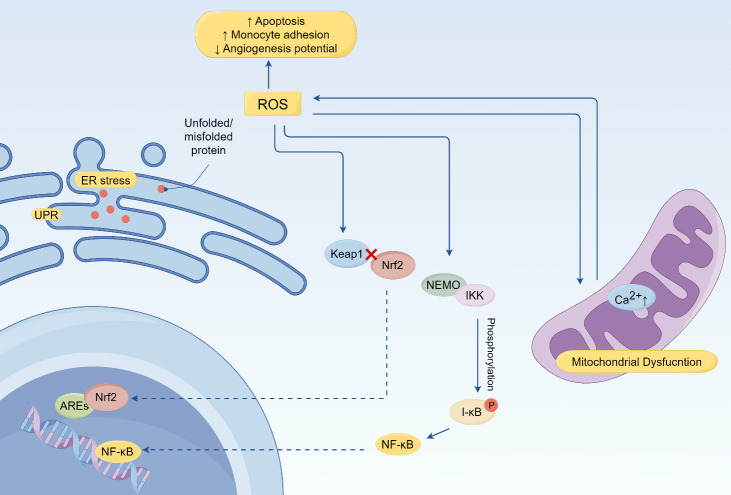
**The effect of ROS in the process of ER stress activation and mitochondrial dysfunction (By Figdraw)**. ER induces ROS production via ERO1 during stress. The release of Ca2+ in the ER causes mitochondrial damage which further aggravates the release of ROS. In the process, ROS, NF-κB, ER stress and mitochondrial dysfunction are interrelated. NF-κB controls the levels of ROS by regulating antioxidant and pro-oxidant genes, and ROS in turn inhibits or enhances the DNA binding activity of NF-κB itself, depending on modifications of NF-κB. ROS also regulates the IKK complex and phosphorylates IκBα.

The endothelium functions as both a source and target of ROS (Fig. 3). ROS induces endothelial dysfunction by causing damage to various organelles, including endoplasmic reticulum stress and mitochondrial impairment. Additionally, oxidative stress is a well-established contributor to endothelial cell senescence, causing dynamic alterations in protein expression, morphology, and metabolism [76].

Firstly, mitochondria play a central role in regulating aerobic energy production and are normally one of the major sources of ROS while also being susceptible to its effects. ROS can disrupt normal calcium homeostasis mediated by mitochondria. For instance, it has been reported that treatment with H_2_O_2_ increases mitochondrial Ca^2+^ concentration in permeabilized cultured endothelial cells, potentially through inhibition of mitochondrial Na^+^/Ca^2+^ exchangers [77].Additionally, mtROS can directly impair mitochondrial function in endothelial cells by targeting electron transport chains and mitochondrial DNA, leading to further generation of excessive ROS [78, 79].

As for endoplasmic reticulum (ER), the ROS and its derivates may result in deactivation of the protective system involving ER chaperones, thereby triggering ER stress and unfolded protein response (UPR). Given that protein folding in ER is highly sensitive to redox states, disruption of disulfide bond formation during ER stress may lead to increased oxidative stress within the ER lumen and impairment of its functionality. For instance, exposure of endothelial to oxLDL led to inactivating modification of PDI, an important ER chaperone responsible for disulfide bond formation [80].

Excessive levels of ROS also exert numerous detrimental effects on the intracellular milieu, including alterations in DNA transcription, disruption of multiple redox-sensitive signaling pathways, and impairment of cellular structure and function [81, 82]. Collectively, these factors contribute to the promotion of endothelial cell senescence. As previously mentioned, Sirtuins constitute a family of signaling proteins with metabolic functions, specifically encompassing deacetylase and ADP-ribosyltransferase activities, H_2_O_2_ induces endothelial cell senescence by downregulating the levels of SIRT6 [22, 75]. *In vitro* experiments also revealed that oxidative stress promote the onset of premature senescence by causing the loss of telomere integrity [83]. Conversely, senescent endothelial cells exhibited an upregulation in the production of endothelin-1 (ET-1) and a downregulation in NO production, thereby facilitating significant intercellular communication with the neighboring cardiac cell population and promoting an atherosclerotic phenotype [84, 85].

In summary, alterations in ROS-producing and ROS-scavenging pathways in vascular endothelium further provoke series of intracellular dysfunction, which could further lead to atherosclerosis.

### 3.4. ROS induces increasing production and accumulation of ox-LDL

It is generally accepted that oxidative ox-LDL plays a more pivotal role in the atherogenic process than native unmodified LDL-C. LDL becomes oxidized to form ox-LDL in pathologic states, which can occur as a result of ROS generation in the arterial wall. Ox-LDL causes endothelial dysfunction, an initial step in the formation of atheroma [86], through activates NADPH oxidases and decreases eNOS activity [87]. In the meantime, the infiltration and accumulation of ox-LDL in the sub-endothelial space is also one of the direct subsequences of endothelial injury.

The retention and subsequent accumulation of LDL in predisposed atherosclerosis area in the vascular wall triggers propagation of lesion development. Due to the local oxidative microenvironment in the sub-endothelial matrix, the LDL particles are more susceptible to generating ox-LDL particles containing bioactive molecules [88]. Ox-LDL, in turn, contributes to the expression of cell adhesion molecules, including like vascular cell adhesion molecules-1 (VCAM-1), P and E- selectins [89]. These initiate the inflammatory response and lead to the excessive production of downstream ROS, which positively regulates the oxidative modification of LDL.

On the other hand, those cell adhesion molecules on the endothelial cells can further lead to leukocyte recruitment into the sub-endothelial space, which is directly linked with the presence of foam cells in the arterial wall [90]. Monocytes originate from bone marrow-derived progenitor cells, and they differentiate to macrophages after tethered on endothelial cells and entering the sub-endothelial space through the interaction of adhesion molecules [91]. One of the main causes of foam cells generation is the excessive influx of ox-LDL in intimal macrophages [92], which can be enhanced by the presence of ROS. In the meantime, newly differentiated further promote the oxidation of LDL, which are then internalized by specific scavenger receptors and giving rise to more foam cells [93, 94]. The release of bioactive proinflammatory lipids subsequent to LDL oxidation, like oxidized phospholipids (OxPL), also exerts both local and systemic actions. The antibody of OxPL has been demonstrated to prevent the inflammatory signaling and decrease atherogenic responses [95]. Furthermore, oxidative stress is an essential cause of advanced lesional macrophage apoptosis. The insufficient autophagy and defective clearance of apoptotic cells promotes plaque necrosis, which precipitates acute atherothrombotic event [96].

LOX-1 is the major receptor involved in the uptake of ox-LDL in endothelial cells [97]. It is widely believed to significantly contribute to the atherogenic process, and its deletion has been demonstrated to effectively reduce atherogenesis in an LDLr-null mice model [98]. LOX-1 is associated with various factors that are involved in the initiation of vascular plaque formation, including the rising transcription of NF-κB, the ascending expression of monocyte chemotactic protein-1 (MCP-1), the downregulation of anti-apoptotic proteins and decreasing transcription of eNOS [99-102]. Studies have shown that exposure to pro-inflammatory cytokines, ox-LDL and free radicals can up-regulate the expression of LOX-1 [103].

### 3.5. ROS jeopardize NO production

Endothelial cells can metabolize L-arginine to form NO and I-citrulline through the endothelial isoform of eNOS [104]. The basal level of NO production contributes to the regulation of vasodilation and prevention of non-thrombogenic behavior in the vascular wall, which is one of the most important atherosclerotic defense mechanisms in the vasculature. Once NO is produced by eNOS, it can rapidly diffuse across cell membranes to act on neighboring smooth muscle cells, circulating platelets and leukocytes. Endothelial-derive NO functions include lowering the vascular resistance, reducing blood pressure, and blood vessel diastole [105].

Thus, the vascular endothelium not only is a monolayer of cells lying between the lumen and the intimal wall, but also modulates blood flow and vascular resistance via the production of NO. In the progression of atherosclerosis and development of endothelial dysfunction, the reduction of bioavailability of NO is regarded as the first key event.

1-Palmitoyl-2-(5-oxovaleroyl)-sn-glycero-3-phosphocholine (POVPC), the oxidation product of oxidized low-density lipoprotein, has been demonstrated to impaired endothelial function and vasodilation by uncoupling and inhibiting eNOS [106]. Similarly, 20-Hydroxyeicosatetraenoic acid and 25-hydroxycholesterol reduce the production of NO and cause endothelial dysfunction also via eNOS uncoupling [107-109].

ADMA causes the reduced eNOS phosphorylation level, which is involved in the mechanism accounting for the decreasing of NO production and endothelial dysfunction [110]. In this process, ROS reduces the activity of dimethylarginine dimethylaminohydrolase (DDAH), a key enzyme in the catabolism of ADMA to induce high level of ADMA and serve as the endogenous inhibitors of eNOS activity [111].

Additionally, under oxidative condition, the decreased concentration of NO can further lead to inhibition of AMPK pathway, delaying the repair of endodermal cells and providing favorable conditions for the formation of atherosclerosis plaque [112]. In the meanwhile, superoxide radical can rapidly react with NO and yield a highly reactive peroxide, OONO^-^, which further combines with eNOS cofactors (such as BH4) and downregulate the bioavailability of NO [113]. Thus, excessive amount of ROS contributes to the turbulence of vascular regulation and accelerated atherosclerosis.

## 4. Biomarkers of oxidative stress

Despite the roles of oxidative stress in many serious diseases have already been demonstrated, measurement of ROS is still difficult. Reactive oxygen species can be assessed directly by electron spin resonance, which has been recognized as the most powerful technique for free radical detection. Also, ROS accumulation level can be measured indirectly by the associated damage to lipids, proteins and nucleic acids. Although direct measurement of ROS is ideal, it is hard for them to accumulate to sufficiently high level to be measured. Thus, indirect methods are more commonly used in laboratory and clinical work.

Various biomolecules can be oxidized by ROS, thus causing structural changes and functional loss. First of all, it is known that lipid oxidation is highly relevant to the process of atherosclerotic cardiovascular diseases. Hydrogel radical (·OH), one of the most reactive and hazardous radicals, plays an important role in lipid oxidation. When hydrogel radical attack lipids containing carbon-carbon double bonds, especially polyunsaturated lipids, an array of oxidation products are generated in sequence. Take arachidonic (20:4) as an example for polyunsaturated fatty acids, which has 3 bis-allylic hydrogens at C7, C10, and C13 positions, thus susceptible to attack by hydroxyl radical and generate endoperoxides [114]. Those endoperoxides subsequently undergo fragmentation to produce a broad range of reactive intermediates. Among them, bioactive aldehydes, such as prostaglandin F2α isomer F2-isoprostanes (F2-IsoPs), 4-hydroxyalkenals, acrolein, and malondialdehyde (MDA), have received particular attention. The assay methods can be divided into two categories, respectively measurement based on concentrations of lipid peroxides themselves, or based on the determination of end oxidative products like MDA [115].

Among all the isoprostanes, 8-isoprostaglandin F2α is the most frequently used marker for measuring oxidative stress. It can be assessed through GC/MS or ELISA kits detection in urinary samples. In a prospective study among 12,239 postmenopausal women over 18 years, the high levels of urinary 8-isoPGF2α were associated with increased incidence of fatal CVD [116].

Similarly, MDA is also one of the most commonly used biomarkers for lipid peroxidation, which is a highly reactive dialdehyde generated from ROS-mediated lipid degradation [117]. Its plasma levels correlate closely with 8-isoPGF2α. Determination of MDA levels often relied on a reaction with thiobarbituric acid (TBA) to generate the reactive products named TBARS, which can be measured through colorimetry of HPLC. MDA serum levels were demonstrated to independently predicted cardiovascular events in patients with coronary artery disease in a prospective randomized evaluation [118].

Ox-LDL is the end product of non-enzymatic oxidative modifications. A meta-analysis of 8644 subjects indicated that increased levels of circulating ox-LDL are associated with clinical stable atherosclerotic cardiovascular disease (ASCVD) events [119]. A prospective population-based survey over 10 years from 40- to 79-year-old men and women suggested that pro-inflammatory oxidized phospholipids, present primarily on ox-LDL, are significant predictors of atherosclerosis [120]. Also, other methods like d-ROMs or Fe-ROMs test can quantify the peroxidation products themselves, providing an assay of measurement and detection of hydroperoxides [121]. Thus, the detection of lipoxidation products can contribute to be the valid biomarkers for the identification of oxidative stress associated with atherosclerosis.

Except from the detection of lipoxidation level, another indicator of oxidative damage is modifications of proteins. The most common indicator of protein oxidation is protein carbonyl content and nitro-tyrosine, which can be detected in serum, plasma and urine samples [122]. A case-control and interventional study of 208 CAD patients revealed that nitro-tyrosine levels are associated with the presence of CAD [123]. Protein carbonyls, the most frequent ROS-induced protein modification generated 2,4-dinitrophenylhydrazine as end product, whose elevated level were detected in CAD patients [124]. Also, advanced glycation end products (AGEs) are protein carbonyls, it activates NF-κB and associated with all-cause and CV mortality [125]. Advanced oxidation protein products (AOPPs) is a result from the interaction between oxidants and plasma proteins, proved to be effective markers of oxidative stress. A multi-center, prospective cohort study of 1394 patients indicated that elevated serum AOPP levels were associated with higher risk of all-cause and CVD mortality [126].

As for nucleic acid, 8-Hydroxy-2′-deoxyguanosine (8-OHdG) and 8-hydroxyguanosine(8-OHG) are sensitive DNA damage marker. A comparative study shows that patients endothelial function is negatively correlated to the 8-OHdG/dG ratio [127].

In addition to oxidatively-modified biomacro-molecules, several other parameters are also used as biomarkers of oxidative stress, like the activities of antioxidant enzymes. MPO is a member of superfamily of heme peroxidases, which indirectly associated with conditions of increased oxidative stress. Several studies have shown a strong correlation between MPO and CVD, including coronary artery disease, congestive heart failure and stroke. A 2001 case-control study showed that blood MPO level was significantly higher in patients with CAD than controls [128]. Additionally, a prospective study included 3375 patients suggested that MPO predicted the risk for CAD with an OR=1.49 [1.20-1.84], P<0.001, showing that MPO level can serve as early indicator of CAD risk [129].

## 5. Pharmacotherapies for ROS-induced endothelial dysfunction

Evidence from experimental and clinical has demonstrated that some clinical used drugs and novel therapy candidates can ameliorate endothelial dysfunction caused by ROS damage. Several clinical trials and meta-analyses have been conducted to evaluate the beneficial effects of antioxidants such as vitamin C, vitamin E, xanthine oxidase inhibitors, N-acetylcysteine (NAC). Also, new promising novel antioxidant approaches like ROS-based nanoparticles have been witnessed, which can load molecular probes to scavenger ROS [7]. In addition, commonly used cardiovascular drugs can also improve multiple aspects of endothelial dysfunction and ROS level.

### 5.1. Broad spectrum antioxidant

As described earlier, oxidative stress is critical for the pathogenesis of atherosclerosis. Thus, the net effect of antioxidant compounds re-establishes the balance between oxidation and reduction to improve endothelial function. Their main mechanisms include inhibition of ROS production, ROS-activated pathway, and lipid peroxidation. Many antioxidants with different structures exert endothelial protective effect, such as vitamin C and E, N-acetylcysteine (NAC), genistein, resveratrol, and melatonin.

Vitamin C and E protect the endothelium by scavenging ROS [130, 131]. A systematic review and meta-analysis enrolled 44 clinical trial showed a significant positive effect of vitamin C on EF in atherosclerotic patients [132]. However, based on a meta-analysis encompassing 294,478 patients in randomized controlled trials, no substantiating evidence was identified to endorse the utilization of vitamin supplements for primary or secondary prevention of major cardiovascular events [133].

Genistein, an isoflavone abundantly present in soybeans, is believed to have protective effect of atherosclerosis, by preventing ox-LDL-induced inflammation. A study based on human umbilical vein endothelial cells showed that genistein could reverse ox-LDL effect through MicroRNA-155/SOCS1-mediated repression of NF-ĸb signaling pathway [134]. A recent study based on human-induced pluripotent stem cells derived oxidative stress model discovered that genistein could bind to the cannabinoid receptor 1 in the vasculature and inhibit its activity, thus attenuates marijuana induced atherosclerosis [135].

N-acetylcysteine (NAC) also presents anti-inflammatory and antioxidative properties. The clinical use of NAC aims to replenishing glutathione stores and indirectly as a precursor for glutathione synthesis [136]. Furthermore, through its antioxidant properties, NAC improves oxygenation and decreases free radical damage by decreasing NF-Κb activation in patients, which also associated with decreases in interleukin-8 [137].

Melatonin exhibits favorable anti-atherosclerotic properties at least partially by enhancing the structure and function of endothelial cells. Its molecular targets encompass cyclophilin A, JNK/Mff signaling, and RORα/miR-223/STAT-3 signaling, thereby contributing to the augmentation of mitochondrial antioxidant enzyme activities, attenuation of reactive oxygen species generation, and maintenance of endoplasmic reticulum homeostasis [138, 139]. A randomized, double-blind trial involved 60 diabetic patients with CHD suggested that melatonin intake (10mg/day) for 12 weeks had beneficial effects on plasma GSH, NO, MDA, PCO, serum hs-CRP levels, glycemic control, HDL-cholesterol, total-/HDL-cholesterol ratio, and blood pressures [140].

Resveratrol, a polyphenolic compound, has been shown in vitro systems to directly scavenge a variety of oxidants, including ·OH, O_2_^·̄^, H_2_O_2_, and OONO^-^. However, the effects of resveratrol against oxidative injury in vivo are more likely relying on gene regulating [141]. Resveratrol enhances the production of endothelial NO, upregulates antioxidant enzymes like SOD, GPx1, and CAT, increases the content of endothelial GSH content and downregulate expression and activity of NOX [141]. Another small, randomized, double-blind, placebo-controlled trial of 56 patients with T2DM and CHD suggested that 4-week supplementation of resveratrol caused a significant increase in total antioxidant capacity (TAC) and a significant reduction in MDA levels [142].

These experimental findings indicate that broad spectrum antioxidants appear to be promising candidates to ameliorate endothelial function and improve the management of cardiovascular diseases. However, despite the potential synergistic effect of surgical and non-surgical treatments in atherosclerosis patients, the translation of basic research findings into a clinical setting still poses challenges for broad spectrum antioxidants.

First, preclinical studies such as animal studies and in vitro experiments are far from representative real biological processes in the human body. Although the antioxidant effect has been well documented, many antioxidants might show no benefit or could be harmful under clinical circumstances. Second, the beneficial effects of vitamin or antioxidant supplements may be related to timing of administration. For example, the beneficial effects of vitamin C occur in the early stages of atherosclerosis. Therefore, despite the favorable data supporting the antioxidant effects of Vitamin C and the anti-atherogenic effects of vitamins C and E, there remains inconsistency in clinical evidence. This discrepancy may be attributed to variations in participant age demographics. Additionally, certain studies propose that antioxidants may confer benefits specifically to individuals exposed to heightened levels of oxidative stress (e.g., diabetics, smokers, and elderly populations), underscoring the significance of targeting specific subgroups for optimal outcomes [143]. In general, further large randomized controlled trials are necessary to evaluate the effectives of antioxidants candidates before wide-scale implementation can be recommended.

## 5.2. Novel therapy

### 5.2.1. ROS based nanoparticles

In addition to broad spectrum antioxidants, novel approaches such as ROS-based nanoparticles (NPs) have demonstrated efficacy in preventing endothelial dysfunction both in laboratory and clinical settings. NPs possess unique characteristics including small size, large surface area, and high potential for biological modification. They not only evade the mononuclear phagocyte system, thereby prolonging their circulation time in the bloodstream, but also exhibit effective targeting of lesions. NPs can be broadly classified into inorganic NPs and organic NPs [144, 145].

Inorganic NPs employed for AS treatment primarily consist of metal nanomaterials. For instance, loading iron oxide nanocarriers with interleukin 10 (IL-10) enables sustained release under mild conditions. Compared to the free IL-10 and PBS groups, NP therapy exhibits significant inhibitory effects on the progression of atherosclerotic plaques. Inorganic NPs with antioxidant properties, such as Mn, Ce, Cu, V, and noble metals provide a novel approach for scavenging ROS [146].

Organic NP carriers are commonly utilized for delivering antioxidants such as andrographolide, berberine, and statins [147-149]. Statin-loaded NPs composed of high-density lipoprotein cholesterol ([S]-rHDL) have been demonstrated to significantly inhibit intracellular ROS production after injection [147]. Moreover, a functional nano-vector encapsulating berberine (BT_1500_M) has been demonstrated to reduce endothelial lesions by modulating AMPK and NF-κB gene expression. Furthermore, certain organic NPs themselves possess intrinsic ROS scavenging capabilities [81, 150].

In general, the development of nanotechnology-based strategies for ROS clearance is anticipated to complement conventional anti-AS clinical agents and offer more precise treatment options for AS. However, certain limitations still exist. Firstly, the preparation of nanoparticles is complicated, and the quality control is difficult. Secondly, extensive *in vitro* and *in vivo* trials of NPs are still required for human patients, encompassing investigations into their long-term side effects, *in vivo* toxicity, drug delivery methods and circulation time [145, 151]. Therefore, the development of a nanotherapeutic integrated platform that is safe, simple, and efficient remains an unmet need.

### 5.2.2. MicroRNA

MicroRNAs are small non-coding RNA at approximately 20 nucleotides in length that regulate gene expression at the post-transcriptional level. They play a key role in both ROS biogenesis and scavenging processes, exhibiting varying expression levels under oxidative stress [38]. The regulation of ROS production by miRNA primarily involves the modulation of molecules within antioxidant pathways, including Nrf2, ARE, and members of the SIRT family [152, 153]. Ongoing clinical trials are investigating the manipulation of miRNAs to target oxidative stress in cardiometabolic disorders, such as miR-210 in peripheral artery disease and miR-133a/miR-208b for acute myocardial infarction. Although clinical studies on miRNA treatment for cardiovascular therapy have been conducted, there is currently no conclusive evidence supporting their standalone therapeutic efficacy [38]. Furthermore, the absence of ongoing clinical trials investigating miRNAs for atherosclerosis necessitates further evaluation of their therapeutic significance. Additionally, it is imperative to explore combination therapies involving multiple miRNAs and develop effective drug delivery methods.

### 5.3. Lipid lowering therapy

Lipid-lowering therapy (LLT) reduces the cardiovascular risk of patients with cardiovascular disease by decreasing blood levels of low-density LDL-C [154]. Before drugs usage, the foundation for managing serum cholesterol is the facilitation of a healthy lifestyle [155]. The improvement of blood lipid levels can be achieved through the maintenance of normal weight and blood sugar levels, as well as the reduction in consumption of simple sugars and refined carbohydrates [156]. A study involving 55685 participants revealed that among participants at high genetic risk, a favorable lifestyle was associated with a nearly 50% lower relative risk of coronary artery disease than was an unfavorable lifestyle [157]. Further, several clinical trials have demonstrated the efficacy of statins, ezetimibe, and proprotein convertase subtilisin/kexin type 9 (PCSK9) inhibitors to confer relative reductions in cardiovascular events.

By inhibiting 3-hydroxy-3-methylglutaryl coenzyme A reductase, statins have been sed for 30 years to prevent coronary artery disease and stroke [158]. It has been proposed that statins exert both LDL-C-dependent and LDL-C-independent effects, also known as “pleiotropic”. The cardiovascular protective effects of statins include improved endothelial function, antioxidant, anti-inflammatory and antithrombotic effects.

One pleiotropic effect observed during statin therapy is the reduction of oxidative stress, the mechanism behind is multifaceted. First, statins display LDL-lowering effect and protective effect against oxLDL, hampering the formation of foam cells. Statins reduce the availability of hepatic cholesterol, which might directly lead to free and esterified cholesterol shortness in apoB-containing lipoproteins. Thereby, the amount of substrate for oxidation directly reduces. On the other hand, statin treatment beneficially alters the atherogenic LDL profile, transforming them to larger and more buoyant particles, which are less susceptible to oxidation [159]. Next, statins can help to increase NO bioavailability by regulating eNOS. As earlier mentioned, the reduction of bioavailability of NO can be regarded as the first key event in atherosclerosis. Simvastatin appears to moderately decreases blood pressure by elevating BH4 production, p-eNOS expression and NO levels in the vascular endothelium [160, 161]. Additionally, simvastatin enhanced phosphorylation of eNOS and increase endothelial progenitor cells via the PI3K/Akt signaling pathway [162]. Furthermore, experimental evidence demonstrated that lovastatin substantially suppressed EndMT and TGF-β1 signaling induced by high glucose in glomerular endothelial cells, exhibiting its ability to inhibit oxidative stress [163]. Thus, statins can ameliorate ROS-induced endothelial dysfuncion.

Meta-analyses of randomized, controlled trials have reported that for every reduction of 1.0 mmol per liter in LDL-C level, statins confer relative reductions in cardiovascular events of 22% [164]. In people at low risk of vascular events, trials focused exclusively on primary prevention of statin also reported similar and significant relative reductions in fatal or nonfatal ASCVD events [165]. For the vast majority of patients treated with statins, the benefits outweigh the risks. The risk of severe adverse event associated with statins is very low, including rhabdomyolysis (<0.1%), serious hepatotoxicity (approximately 0.001%) [166]. Statins exhibit a greater efficacy in reducing the risk of atherothrombotic stroke and, consequently, total stroke incidence, thereby counterbalancing the potential increase in hemorrhagic stroke risk among patients with cerebrovascular disease [166].

While the clinical benefit of lowering LDL-C with statins remains widely accepted, recent American College of Cardiology expert consensus document recommended considering adding certain non-statin therapies to lower LDL-C, including PCSK9 inhibitors, bempedoic acid and ANGPTL3 inhibitors [167-170].

The PCSK9 enzyme binds to the LDL receptor, facilitating its internal hepatic degradation, and gain-of-function mutations in this process are implicated in familial hypercholesterolemia [171]. PCSK9 inhibitors are subcutaneously injected monoclonal antibodies that inactivate the PCSK9 enzyme. The ODYSSEY Outcomes (Evaluation of Cardiovascular Outcomes After an Acute Coronary Syndrome During Treatment With Alirocumab) trial, demonstrating the benefits of alirocumab in patients with acute coronary syndrome with dramatic reduction in LDL-C levels of approximately 60% [172]. In 2017, the FOURIER (Further Cardiovascular Outcomes Research with PCSK9 Inhibition in Subjects with Elevated Risk) trial, involving 27,564 patients with atherosclerotic cardiovascular disease, has demonstrated that inhibition of PCSK9 with evolocumab on a background of statin therapy was efficacious in patients. Evolocumab could lowered LDL-C levels from 70 mg per deciliter or higher to a median of 30 mg per deciliter and reduced the risk of cardiovascular events [173]. Thus, based on evidence from IMPROVE-IT (IMProved Reduction of Outcomes: Vytorin Efficacy International Trial), FOURIER, and ODYSSEY Outcomes, the addition of ezetimibe, evolocumab, and alirocumab exhibit clear cardiovascular benefit to high-risk patients with ASCVD [167, 172-174]. However, safety beyond 3 years is not yet well established, and the addition of PCSK9 inhibitors to statin therapy has a low probability (<1%) of being cost effective until the annual cost drop 62%, to $5459 per year [175].

### 5.4. Anti-hypertensive drugs

The renin-angiotensin-aldosterone system (RAAS) is an important part of inflammatory and oxidative response regulation, exerting crucial impact on atherogenesis. Many of the atherogenic effects of Angiotensin II, such as endothelial dysfunction, cellular proliferation, and inflammation itself are mediated by impaired NO synthesis and ROS production [176].

Antihypertensive drugs such as angiotensin receptor blockers (ARBs), calcium channel blockers (CCBs), and ACEI have shown multiple protective actions in ameliorating endothelial dysfunction. Clinical evidence has shown that treatment with ARBs, CCBs. and ACEIs may improve endothelial function in patients at high risk of cardiovascular events [176]. Mechanically, those antihypertensive drugs relief ROS damage in endothelium by reducing NOX4 level, enhancing eNOS phosphorylation, and recoupling eNOS to increase NO bioavailability [176].

Aside from humoral factors, the chronic sympathetic hyperactivity can increase circulating norepinephrine and mitochondrial monoamine oxidase A (MAO-A) activity, enhancing oxidative stress to induce endothelial dysfunction. Thus, renal denervation is a strategy to reduce sympathetic tone, ameliorating mitochondrial ROS-induced inflammation to reduce atherosclerosis [177]. A study on ApoE deficient (ApoE ^-/-^) mice suggested that levels of oxidative stress indicators in mouse serum, such as SOD, MDA, and T-AOC, were markedly decreased, while GSH levels increased significantly after renal denervation [177].

## Conclusions

This article provides a comprehensive overview of the fundamental definition of ROS, molecular functions, antioxidant systems, as well as their relationship with endothelial dysfunction and atherosclerosis. Additionally, it comprehensively examines the four primary sources of ROS in endothelial cells and elucidates the intricate molecular mechanisms, by which ROS contributes to endothelial dysfunction in the pathogenesis of atherosclerosis. Additionally, this review presents both in vitro and in vivo evidence supporting the protective effects of oxidative stress-targeted interventions on atherosclerosis-associated endothelial dysfunction. While numerous studies have demonstrated the efficacy of antioxidants in improving cardiovascular function, clinical trial evidence remains controversial; thus, necessitating further large-scale randomized controlled trials. Moreover, future clinical studies should explore novel approaches such as ROS-based nanoparticles (NPs) or multiple targets-targeted antioxidant strategies.
